# Letter regarding “Puppyhood diet as a factor in the development of owner‐reported allergy/atopy skin signs in adult dogs in Finland”

**DOI:** 10.1111/jvim.16498

**Published:** 2022-07-22

**Authors:** Brennen McKenzie, Jennifer Larsen

**Affiliations:** ^1^ Adobe Animal Hospital Los Altos California USA; ^2^ Department of Veterinary Medicine University of California, Davis Davis California USA

Dear Editors,

The report Puppyhood diet as a factor in the development of owner‐reported allergy/atopy skin signs in adult dogs in Finland, which appeared in the September/October issue of the *Journal of Veterinary Internal Medicine* (*JVIM*), suggests that “puppyhood exposure to raw animal‐based foods,” “human meal leftovers” and other “real foods” might have a protective influence against canine atopic dermatitis (CAD) while “heat‐processed foods” might increase later occurrence of CAD. We are concerned that the results of this study likely represent residual bias in data collection and analysis rather than the actual influence of specific dietary components on the risk of CAD.

Common recognized sources of bias in research studies include a priori beliefs on the part of researchers, recall and selection bias in survey respondents,^1^ funding bias,^2^ and a large number of researcher degrees of freedom in the design, conduct, and analysis of the study.^3^ All of these are present in this study.

Dr. Hielm‐Björkman and the Dog Risk research group have expressed strong beliefs about the health benefits of raw diets and the dangers of conventional commercial pet foods in previous research reports and popular media.^4^, ^5^

The main source of data in this study is an online survey available only in Finnish. Previous publications regarding validation of this data source have identified low response rates to validation questions and important proportions of duplicate, automated, and discordant responses.^6^, ^7^ Asking owners to remember in detail what they fed their puppy between 2 and 8 months of age and then trying to associate that with health outcomes years later is, in our view, a questionable strategy. Self‐reporting of diet and health information are unreliable in humans, and we believe that it is unlikely to be more reliable among dog owners.^1^ Dog owners consistently misperceive even straightforward measures such as body condition score despite formal training in this assessment, so their assessment of signs of CAD is, in our view, likely to be equally unreliable.^8^ The survey responses are expressions of the perceptions and beliefs of the owners who participated, not necessarily the true nutritional and environmental exposures nor health outcomes experienced by the dogs.

Dr. Hielm‐Björkman has previously cited funding bias as a problem in the study of raw diets, stating that raw foods are “not really researched in universities. Most universities get sponsored by these big billion‐dollar companies, and you don't really want to step on their toes, I guess.”^5^ In light of the recognized problem of funding bias it could be relevant that disclosed funding sources for this research include companies selling raw pet foods.

The authors also report accepting funding from Dr. Joseph Mercola. Dr. Mercola's organization advocates for raw pet diets and argues against conventional pet foods on its web site.^9^, ^10^, ^11^ We believe that he is also a consistent promoter of unapproved medical practices and medical product claims, as evidenced by multiple warning letters from the Food and Drug Administration and a lawsuit by the Federal Trade Commission related to therapeutic claims that violate the U.S. Food, Drug, and Cosmetic Act.^12^, ^13^, ^14^, ^15^, ^16^ Such an affiliation is at least as relevant to assessing potential bias as accepting funding from an organization with a commercial interest in raw or conventional pet foods. Bias in scientific research is as likely to arise from ideological as financial factors.

We believe that the results of the analysis and how they are reported also suggest the influence of bias. Many comparisons are listed only in supplemental materials, and those reported in the main article tend to be those which support the authors' hypotheses. This creates the appearance of consistency when, in fact, the associations identified are often inconsistent and do not logically support the claims in the conclusions. For example, why would raw tripe and organ meats be protective against CAD but raw red meat, eggs, and poultry not be? If cooking is the key risk factor, why would cook vegetables be protective and raw vegetables would not or why would both cooked and raw eggs be protective while neither cooked nor raw poultry is associated with the likelihood of CAD? If exposure to bacteria is the main variable, why is eating dirt, sticks or carcasses protective but eating clay and grass is not and drinking from puddles is actually associated with increased risk? If excessive processing is the issue, why was there no association with eating processed meats or canned foods and only a marginal association with dry food when it was the only food offered?

Dogs with CAD were reportedly more likely to be eating no raw food at all than controls, and dogs without CAD were more likely to be fed 20% or 90% raw, but there were no statistically significant differences at any other ratio of the 2 foods. Similarly, allergic dogs were more likely to be fed 80% dry than controls, but there was no significant difference in CAD risk if they were fed more than 80% dry. Control dogs were more likely to be fed 50% or less than 10% dry, but there was no difference at intermediate ratios. It is easier to see cherry picking and researcher degrees of freedom than a consistent dose‐response in these results.

While the authors do acknowledge the potential for recall bias and misclassification, and they do state that their findings cannot prove causal relationships, the overall message of the paper is that uncooked foods and human meal leftovers likely have health benefits, and this is inconsistent with the methodological limitations and potential for residual bias of this study.

Given the importance of CAD, in terms of both prevalence and the negative impacts on pet and owner quality of life, we agree that identification of protective factors is warranted. However, considering the significant risks of illnesses and death that have been consistently associated with the feeding of raw animal products, rigorous and well‐designed research methodology, objectivity, and full reporting of results are needed to explore if any benefits exist for this practice, and certainly are required before this practice can be confidently recommended.

## CONFLICT OF INTEREST DECLARATION

Jennifer Larsen declares the following conflicts:

- Investigator in clinical trials and other research partly or fully sponsored by Royal Canin, Nature's Variety Instinct, and Nestle Purina PetCare.
- Develops educational materials for Mark Morris Institute and HealthyPet magazine.
- Serves as advisory group consultant for Elanco Animal Health.
- Participates in continuing education events, as a speaker & as an attendee, sponsored/organized by Royal Canin, Nestle Purina PetCare, Nature's Variety Instinct, and Hill's Pet Nutrition.
- A resident of the Nutrition Service received funds through the Hill's Pet Nutrition Resident Clinical Study Grants program.
- The Veterinary Medical Teaching Hospital at the University of California, Davis receives funds from Nestlé Purina PetCare to partially support a nutrition technician.

Brennen McKenzie declares no conflict of interest.
